# Assessment of hip involvement in patients with ankylosing spondylitis: reliability and validity of the Hip Inflammation MRI Scoring System

**DOI:** 10.1186/s12891-021-04502-3

**Published:** 2021-08-17

**Authors:** Siliang Man, Liang Zhang, Tao Bian, Hongchao Li, Zhuyi Ma, Yixin Zhou

**Affiliations:** 1 Department of Rheumatology, Beijing Jishuitan Hospital, Fourth Clinical College of Peking University, No. 31 Xinjiekou East Street, Xicheng District, 100035 Beijing, China; 2 Department of Orthopedics, Beijing Jishuitan Hospital, Fourth Clinical College of Peking University, No. 31 Xinjiekou East Street, Xicheng District, 100035 Beijing, China

**Keywords:** Ankylosing spondylitis, Hip, Classification system, Reliability

## Abstract

**Background:**

This study aimed to test the reliability and validity of the Hip Inflammation MRI Scoring System (HIMRISS) in assessing hip involvement of AS patients with AS at different stages of the bath ankylosing spondylitis radiology index (BASRI-hip) scoring system.

**Methods:**

Fifty-two outpatients with ankylosing spondylitis (AS) were included in this study. The subjects’ data includes demographics, clinical characteristics, disease activity score, and functional index. Based on the Harris hip scoring (HHS) of involved hip and BASRI-hip score, we devided these patients into no hip involvement group((HHS ≥ 80 and BASRI ≤ 1) (Group A), mild hip involvement subgroup (BASRI = 2 or BASRI ≤ 1 and HHS ≤ 79) (Group B), and moderate to advanced hip involvement subgroup (BASRI ≥ 3) (Group C). Data was analyzed statistically by SPSS software.

**Results:**

In total of 44 patients (88 hips), group A consisted of 21 hips, group B consisted of 42 hips and group C consisted of 25 hips. The test–retest intraclass correlation coefficients (ICCs) in four raters were 0.955 ~ 0.977 and interrater ICC was 0.993. HIMRISS correlated moderately with the Bath Ankylosing Spondylitis Disease Activity Index (BASDAI) (r = 0.540, p < 0.001), the Bath ankylosing spondylitis functional index (BASFI) (r = 0.540, p < 0.001), the Bath Ankylosing Spondylitis Functional Index (BASFI) (r = 0.581, p < 0.001), ASDAS-ESR (r = 0.604, p < 0.001), and Ankylosing Spondylitis Disease Activity Score (ASDAS)-C reactive protein (CRP) (r = 0.575, p < 0.001). HIMRISS in groups B and C was significantly higher than that in group A: 29.38 (17.00, 40.94) vs. 14.50 (11.38, 22.25), p = 0.009; 38 (31.13, 64.38) vs 14.50 (11.38, 22.25), p < 0.001.

**Conclusions:**

HIMRISS applied to patients with AS demonstrated a satisfactory reliability, meaning it is a reliable quantitive assessment tool for evaluating early hip involvement in patients with AS.

## Introduction

Hip involvement is common in patients with ankylosing spondylitis (AS) [1–3] and is associated with severe functional impairment, work disability, a compromised psychological status and quality of life [4–6]. When hip involvement progresses to an advanced symptomatic stage, total hip arthroplasty (THA) which is acknowledged as the well-accepted treatment currently will be conducted, and several authors have reported their results using cemented or cementless techniques [7–12]. Unfortunately, hip prostheses with various exclusive designs have a limited life span, and revision surgeries are often needed periodically. Consequently, it is extremely crucial to establish a comprehensive and reliable system for accurate and early diagnosis of hip involvement in AS patients.

In previous studies, the diagnosis of hip involvement has been focused on clinical findings and radiographic changes. The symptom of hip involvement is an insidious onset, and there are even no symptoms in the early course of the disease. The bath ankylosing spondylitis radiology index (BASRI-hip), which has been proved to be an objective and reliable grading system is the most widely used radiographic classification system to assess severity and progression of hip involvement in patients with AS [13–16]. Conventional radiographs can display post-inflammatory structural injuries and cannot reflect some key histopathological changes, including subchondral bone marrow edema (BME) and synovitis [17, 18]. Consequently, radiographs may underestimate the frequency of hip involvement leading to a diagnosis delay.

Magnetic resonance imaging (MRI) provides excellent visualization of bones and soft tissues and is the only imaging tool capable of visualizing bone marrow inflammation, a hallmark of AS [19, 20]. In previous studies, however, the application of MRI imaging is mainly focused on sacroiliac joints and spine of patients with AS. There is few data of the changes of hip MR imaging [21–23]. The Hip Inflammation MRI Scoring System (HIMRISS) was developed using the data from a trial of intraarticular steroid therapy for osteoarthritis(OA), which is based on the concept that scoring of hip OA most usefully emphasizes the evidence of active inflammation [24, 25]. The feasibility and the reliability of this imaging system have been fully validated in the setting of hip OA. Zheng et al. introduced HIMRISS into the evaluations of spondyloarthritis (SpA), and the reliability and the clinical association were preliminarily validated as being efficient [26]. However, this study failed to give a precise definition of clinical or radiographic hip involvement to these patient population. This study aimed to test the reliability and validity of the HIMRISS for assessing hip involvement in patients with AS at different stages of the BASRI-hip scoring system.

## Materials and methods

### Patient demographics and enrollment

Fifty-two AS outpatients who were admitted to the department of rheumatology and the department of adult joint reconstruction surgery in our institute from February 2018 to June 2019 were enrolled inthis study. The inclusion criteria were as follows: (1) diagnoses of AS were made according to the 1984 modified New York criteria [27]; (2) the age of the patients ranged from 18 to 45 years. Exclusion criteria that any selected patients with any of the followings: (1) systemic diseases of the muscular of nervous systems, (2) history of congenital or childhood disease, surgery, deep infection, trauma, and tumor of hip; (3) lower extremity replacement or amputation other hip joint; (4) MRI contraindications (e.g. pacemaker, metal implants, pregnancy, claustrophobia).

The subjects’ demographics and clinical characteristics included gender, body mass index (BMI), age at outpatient visit, age at onset of AS, duration of AS, diagnosis delay, family history, medication status and extra-articular manifestations (EAMs) (current or past) including uveitis, psoriasis, and inflammatory bowel disease (IBD). Disease activity was assessed respectively using the Bath Ankylosing Spondylitis Disease Activity Index (BASDAI) [28]and Ankylosing Spondylitis Disease Activity Score (ASDAS) [29]. The functional status was assessed using the Bath ankylosing spondylitis functional index (BASFI) [30]. The patient-reported outcomes (PROs) were assessed by using the Ankylosing Spondylitis Quality of Life (ASQoL) scales [31] and the short form-12(SF-12) [32]. The use of the medications including NSAIDs and DMARDs was recorded, and the patients who had taken treatment agents for 12 months or longer were considered as sustained users. These data for clinical characteristics were collected and evaluated independently by two rheumatologists (L.H.C. and M.S.L.) who had not participated in radiographic evaluations conducted from a face-to-face questionnaire and medical records. The Harris hip scoring (HHS) system [33] was directly evaluated by orthopedic surgeons (Z.L. and H.Y.) at the time of outpatient consultation. On a 100-point scale, a score of ≥ 90 points is defined as an excellent outcome, 80–89 points, a good outcome, 70–79 points, a fair outcome, and ≤ 70 points, a poor outcome.

Laboratory data such as human leukocyte antigen (HLA-B27) status, serum erythrocyte sedimentation rate (ESR), high sensitive C reactive protein (CRP) and CRP level were also measured at enrollment.

The results of these clinical and laboratory parameters are provided in Table 1.

**Table 1:** Patient demographics, clinical and laboratory parameters of AS patients on different stages of hip involvement

	Total AS patients	Group A (*n* = 21)	Group B (*n* = 42)	Group C (*n* = 25)	*P* value	Adjusted *P* value		
						A-B	A-C	B-C
Male gender, n (%)	72 (81.8%)	20 (95.2%)	33 (78.6%)	19 (76.0%)	0.175			
Age at outpatient visit (years)	30.00 (25.25, 34.75)	32.00 (30.00, 39.00)	30.00 (25.50–34.25)	28.50 (25.00–35.00)	0.147			
Age at onset (years)	23.00 (19.00, 26.00)	25.00 (23.00, 28.00)	22.00 (18.00, 27.25)	21.50 (17.00, 24.00)	0.005^*^	0.093	0.004^*^	0.390
Disease duration (years)	6.50 (2.00, 10.50)	6.00 (2.00, 11.00)	6.00 (2.00, 9.00)	7.00 (2.00, 11.00)	0.812			
Diagnosis delay (years)	2.00 (0.25, 8.00)	1.00 (0.00, 6.00)	2.00 (0.00, 8.00)	2.00 (1.00, 10.50)	0.233			
Family history, n (%)	27 (30.7%)	6 (28.6%)	9 (21.4%)	12 (48.0%)	0.081			
EAMs, n (%)	14 (15.9%)	1 (4.8%)	7 (16.7%)	6 (24.0%)	0.213			
Uveitis, n (%)	3 (3.4%)	0 (0.0%)	0 (0.0%)	3 (12.0%)	0.033^*^	\	0.239	0.048^*^
IBD, n (%)	7 (8.0%)	1 (4.8%)	5 (11.9%)	1 (4.0%)	0.595			
Psoriasis, n (%)	4 (4.4%)	0 (0.0%)	2 (4.8%)	2 (8.0%)	0.570			
Use of NSAIDs, n (%)	82 (93.2%)	19 (90.5%)	39 (92.9%)	24 (96.0%)	0.327			
Use of DMARDs, n (%)	36 (40.9%)	8 (38.1%)	17 (40.5%)	11 (44.0%)	0.922			
BMI	25.95 (22.86, 29.09)	27.12 (23.12, 29.93)	25.81 (22.86, 29.36)	25.95 (19.59, 28.98)	0.752			
HLA-B27 positivity, n (%)	80 (90.9%)	21 (100.0%)	38 (90.5%)	21 (84.0%)	0.165			
ESR (mm)	25.50 (13.00, 38.00)	13.00 (10.00, 24.50)	25.00 (13.00, 39.25)	29.00 (25.00, 41.25)	< 0.001^*^	0.016^*^	< 0.001^*^	0.296
CRP (mg/L)	14.80 (9.30, 27.80)	13.20 (6.60, 15.56)	14.30 (5.28, 27.80)	24.20 (12.70, 53.80)	0.003^*^	0.610	0.003^*^	0.032^*^
hsCRP(mg/L)	13.54 (4.60, 28.17)	7.70 (4.93, 20.44)	12.20 (3.42, 29.49)	25.96 (8.83, 34.24)	0.031^*^	0.869	0.031^*^	0.179
ALB (g/L)	45.50 (43.00, 47.45)	44.90 (41.30, 46.80)	46.30 (43.95, 47.70)	43.90 (43.00, 46.65)	0.134			
HGB(g/L)	142.00 (133.00, 150.00)	145.00 (141.00–159.00)	143.00 (131.50–152.00)	141.00 (130.00–149.00)	0.123			
BASDAI	3.20 (1.80, 5.35)	2.00 (1.40, 3.00)	3.40 (1.65, 5.25)	4.60 (3.20, 5.80)	< 0.001^*^	0.070	< 0.001^*^	0.023^*^
BASFI	1.60 (0.73, 2.60)	0.70 (0.60, 1.30)	1.80 (0.80, 2.90)	2.00 (1.60, 2.83)	< 0.001^*^	0.001^*^	< 0.001^*^	0.214
ASQOL	6.00 (2.00, 8.00)	2.00 (2.00, 3.00)	6.00 (2.75, 8.00)	7.00 (6.00, 11.75)	0.001^*^	0.011^*^	0.001^*^	0.541
ASDAS-ESR	2.35 (1.83, 3.10)	1.79 (1.05–1.96)	2.44 (1.90–3.48)	2.93 (2.35–3.55)	< 0.001^*^	< 0.001^*^	< 0.001^*^	0.443
ASDAS-CRP	2.45 (1.61, 3.23)	1.54 (1.43, 1.98)	2.52 (1.79, 3.46)	2.63 (2.37, 3.42)	< 0.001^*^	< 0.001^*^	< 0.001^*^	0.538
SF-12PCS	42.20 (27.70, 52.20)	50.10 (39.00, 55.40)	42.20 (28.38, 50.40)	32.10 (22.80, 51.83)	0.020^*^	0.092	0.020^*^	1.000
SF-12MCS	42.75 (27.50, 53.50)	52.20 (40.00, 54.75)	42.35 (29.45, 53.50)	31.90 (25.40, 51.75)	0.038^*^	0.279	0.032^*^	0.675
HHS	86.00 (67.00, 94.75)	95.00 (93.00, 96.00)	77.00 (54.00, 89.75)	69.00 (51.50, 87.00)	< 0.001^*^	< 0.001^*^	< 0.001^*^	0.875
BARSI	2.00 (1.00, 3.00)	1.00 (1.00, 1.00)	2.00 (1.00, 2.00)	3.00 (3.00, 4.00)	< 0.001^*^	0.002^*^	< 0.001^*^	< 0.001^*^
HIMRISS	29.63 (15.25, 43.00)	14.50 (11.38, 22.25)	29.38 (17.00, 40.94)	38 (31.13, 64.38)	< 0.001^*^	0.009^*^	< 0.001^*^	0.103

^*^P < 0.05, *AS* ankylosing spondylitis, *EAMs* extra-articular manifestations, *IBD* inflammatory bowel disease, *NSAIDs* nonsteroidal anti-inflammatory drugs, *DMARDs* disease modifying anti-rheumatic drugs, *BMI* bone mass density, *HLA-B27* human leucocyte antigen-B 27, *ESR* erythrocyte sedimentation rate, *CRP* C reactive protein, *hsCRP* high sensitive C reactive protein, *ALB* albumin, *HGB* hemoglobin, *BASDAI* Bath ankylosing spondylitis disease activity index, *BASFI* Bath ankylosing spondylitis functional index, *ASQOL* ankylosing spondylitis quality of life, *ASDAS* Ankylosing Spondylitis Disease Activity Score, *SF-12 PCS* short form-12 physical component summary, *SF-12 MCS* short form-12 mental component summary, *HHS* Harris hip score, *BASRI-Hip* the bath ankylosing spondylitis radiology hip index

Group A represents the hips with no hip involvement (HHS ≥ 80 and BASRI ≤ 1), group B represents the hips with mild hip involvement (BASRI = 2 or BASRI ≤ 1 and HHS ≤ 79) and Group C represents the hips with advanced hip involvement (BASRI ≥ 3)

The value of continuous variables was presented as median and quartile (25–75%) and the categorical variables were based on presented as number plus percentage

### Radiographic classification system

The anteroposterior (AP) radiographs of the pelvis and MRI images were obtained on the same day on an outpatient basis. We excluded eight patients with inadequate radiograph quality and reserved a total of 44 patients for review and analysis.

The BASRI hip system was adopted to assess the severity of radiological involvement in the hip joint [13], and it classified the status of the hip joints into a five-point scale from 0 to 4 (0 = normal, no change; 1 = suspicious, possible focal joint space narrowing; 2 = minimal, circumferential joint space narrowing > 2 mm; 3 = moderate, circumferential joint space narrowing ≤ 2 mm, or bone-on-bone apposition of ≤ 2 cm; 4 = severe, bone deformity or bone-on-bone apposition of < 2 cm or total hip re-placement).

MRI scans on enrolled patients’ both hips were performed on a 3.0 T MR system (Siemens MAGNETOM Skyra, Siemens Company) using the following imaging parameters: a transverse T1-Weighted Spin Echo (T1 TSE) sequence, a transverse T2 TSE + Fat-Sat sequence, a coronal T1 TSE sequence, a coronal T2 TSE + Fat-Sat sequence, and transverse and coronal fat-saturated. All sequences used a 380 mm field of view, a 3.5 mm slice thickness, a 0.7 mm slice interval, a 384 448 matrix, and several excitations (AVERAGE) of 2.

HIMRISS has three features: bone marrow lesions (BMLs), synovitis and effusion. BML is defined as an area of hyper‐intensity within the bone in STIR sequence. The BML scoring on either femoral head or acetabulum side was graded according to the method which was described by Maksymowych et al. [34]. The range of BML scoring is 0–100. Effusion and synovitis are scored together depending on the maximum depth of the fluid (0 = –0 to 1.9 mm, 1 = –2 to 3.9 mm, 2 =  ≥ 4 mm) on the same central, anterior and posterior slices. The range of total effusion scoring is 0‐30. Therefore, the final HIMRISS scoring range is 0‐130.

### Reading exercises

Considering the possible effect of the raters’ experiences on evaluating the hips, we chose four raters with different levels of training and experiences. A senior rheumatologist (L.H.C.) who is an attending physician with 16 years of clinical experience, an adult hip surgeon (Z.L.) who is a vice director with 15 years of clinical experience, and two musculoskeletal radiologists (Z.J., X.P.) who are residents with 4 years and 9 years of clinical experience respectively participated in grading the MRI imaging.

None of the raters was formed about patient demographics and clinical parameters. First, they learned the scoring rule through a PowerPoint file of Outcome Measures in Rheumatology (OMERACT) 11 [24] individually. Then a training session was held where they agreed on a criteria for radiographic evaluation that was based on five MRI images.

Next, another 88 MRI images from the PACS (Picture Archiving and Communication System) workstation which were not included in the evaluation were collected by the first author (M.S.L.) and were sent to the raters. Then they graded the MRI images in random sequences in different workrooms (exercise 1). Three months later, they repeated their works (exercise 2) without knowing the previous results to assess the test–retest reliability.

### Statistical analysis

Data were statistically analyzed using SPSS software for Windows (version 23.0; IBM, Armonk, NY, USA). Descriptive analyses for categorical variables were shown as percentages and frequencies. Moreover, for continuous variables, they were based on mean and standard deviation (SD) or median and quartile (25–75%) if the data were skewed. The values of femoral BML, acetabular BML, synovitis effusion were summed up as the HIMRISS values for the single hip, and the mean values by all readers obtained in exercise 2 were taken as the final HIMRISS scores. The inter-rater and intra-rater reliability of HIMRISS were calculated using intraclass correlation coefficient (ICC). The correlations of HIMRISS with clinical continuous variables and with ordinal variables were determined by correlation coefficient ® in Pearson correlation analysis and Spearman rank correlation analysis respectively. We classified hips into no hip involvement group(HHS ≥ 80 and BASRI ≤ 1) (Group A), mild hip involvement subgroup (BASRI = 2 or BASRI ≤ 1 and HHS ≤ 79) (Group B), and moderate to advanced hip involvement subgroup (BASRI ≥ 3) (Group C) based on the HHS of involved hip and BASRI-hip score. Demographic features, clinical characteristics and radiographic parameters were compared using ANOVA (including post hoc analysis) and nonparametric Kruskal–Wallis test among these subgroups. Bonferroni method was taken to adjust the significance level in multiple comparisons. All reported P values were two-tailed with an alpha of 0.05.

### Ethics and registration

All procedures involving human participants carried out in the studies were in accordance with the ethical standards of the institutional and/or national research committee and the 1964 Helsinki declaration and its later amendments or comparable ethical standards. This study was approved by the Beijing Jishuitan Hospital Institutional Review Board (project number S-305/2007), and the informed consent was obtained from each participant before the enrollment of this study.

## Results

### Patient demographics and clinical parameters

The patient demographics and clinical parameters were displayed in Table 1. The results of BASRI-Hip score were as follows: 1 score in 33 hips (37.5%), 2 in 30 hips (34.1%), 3 in 18 hips (20.5%) and 4 in 7 hips (8.0%). The HHS was excellent for 30 hips (34.1%), good for 18 (20.5%), fair for 11 (12.5%), and poor for 29 (33.0%). Consequently, group A consisted of 21 hips, group B consisted of 42 hips and group C consisted of 25 hips.

### Reliability of HIMRISS scores

The test–retest and interrater ICCs were provided in Table 2. The interrater ICCs were calculated by four raters in the second scoring exercise.

**Table 2:** The test–retest and interrater reliability of HIMRISS scores in AS patients (*N* = 88)

	ICC	95% CI
Test–retest		
Rheumatologist	0.977	0.965–0.985
Surgeon	0.973	0.956–0.983
Radiologists 1	0.959	0.938–0.973
Radiologists 2	0.955	0.932–0.970
Intrarater		
All four raters	0.993	0.990–0.995

*HIMRISS* Hip inflammation MRI scoring system, *ICC* intraclass correlation coefficient, *95% CI* 95% confifidence interval

### Correlation analysis and intergroup comparisons

The results of correlations between HIMRISS and the clinical and laboratory parameters were shown in Table 3. HIMRISS correlated moderately with BASDAI (*r* = 0.540, *p* < 0.001), BASFI (*r* = 0.581, *p* < 0.001), ASDAS-ESR (*r* = 0.604, *p* < 0.001), and ASDAS-CRP (*r* = 0.575, *p* < 0.001). HIMRISS correlated weakly with ASQOL (*r* = 0.478, *p* < 0.001), ESR (*r* = 0.426, *p* < 0.001), CRP (*r* = 0.449, *p* < 0.001), hsCRP (*r* = 0.398, *p* < 0.001) and HHS (*r* = -0.468, *p* < 0.001).

**Table 3:** Correlations between HIMRISS score and clinical and laboratory parameters

	Correlation coefficient	*P* value
Age at outpatient visit (years)	-0.161	0.134
Age at onset (years)	-0.205	0.055
Disease duration (years)	0.022	0.841
Diagnosis delay (years)	0.091	0.398
Family history, n (%)	0.163	0.129
EAMs, n (%)	0.113	0.299
Uveitis, n (%)	0.154	0.152
IBD, n (%)	-0.023	0.831
Psoriasis, n (%)	0.093	0.387
Use of NSAIDs, n (%)	0.068	0.527
Use of DMARDs, n (%)	0.005	0.966
BMI	-0.137	0.203
HLA-B27 positivity, n (%)	-0.167	0.119
ESR (mm)	0.426	< 0.001^*^
CRP (mg/L)	0.449	< 0.001^*^
hsCRP(mg/L)	0.398	< 0.001^*^
ALB (g/L)	-0.167	0.120
HGB(g/L)	-0.267	0.012^*^
BASDAI	0.540	< 0.001^*^
BASFI	0.581	< 0.001^*^
ASQOL	0.478	< 0.001^*^
ASDAS-ESR	0.604	< 0.001^*^
ASDAS-CRP	0.575	< 0.001^*^
SF-12PCS	-0.270	0.011^*^
SF-12MCS	-0.222	0.037^*^
HHS	-0.468	< 0.001^*^

^*^P < 0.05, *HIMRISS* Hip inflammation MRI scoring system, *EAMs* extra-articular manifestations, *IBD* inflammatory bowel disease, *NSAIDs* nonsteroidal anti-inflammatory drugs, *DMARDs* disease modifying anti-rheumatic drugs, *BMI* bone mass density, *HLA-B27* human leucocyte antigen-B 27, *ESR* erythrocyte sedimentation rate, *CRP* C reactive protein, *hsCRP* high sensitive C reactive protein, *ALB* albumin, *HGB* hemoglobin, *BASDAI* Bath ankylosing spondylitis disease activity index, *BASFI* Bath ankylosing spondylitis functional index, *ASQOL* ankylosing spondylitis quality of life, *ASDAS* Ankylosing Spondylitis Disease Activity Score, *SF-12 PCS* short form-12 physical component summary, *SF-12 MCS* short form-12 mental component summary, *HHS* Harris hip score, *BASRI-Hip* the bath ankylosing spondylitis radiology hip index

Group A represents the hips with no hip involvement (HHS ≥ 80 and BASRI ≤ 1), group B represents the hips with mild hip involvement (BASRI = 2 or BASRI ≤ 1 and HHS ≤ 79) and Group C represents the hips with advanced hip involvement (BASRI ≥ 3)

The value of continuous variables was presented as mean ± standard deviation and the categorical variables were based on presented as number plus percentage

The results of intergroup comparisons were provided in Table 1. HIMRISS of group B and group C was significantly higher than that of group A: 29.38 (17.00, 40.94) vs. 14.50 (11.38, 22.25), *p* = 0.009; 38 (31.13, 64.38) vs. 14.50 (11.38, 22.25), *p* < 0.001. Interestingly, there was no statistical significance (*p* = 0.103) in the difference of HIMRISS between group B and group C.

## Discussion

HIMRISS as a quantitative imaging method possess outstanding advantages in the assessment of early hip involvement in AS compared with the traditional MRI descriptive diagnosis. As far as we know, this was the first study which introduced the HIMRISS into this field. Although HIMRISS was first developed by rheumatologists and radiologists to evaluate the severity of hip involvement in patients with hip OA, the items and scoring details of this system, including bone marrow lesions (BMLs), synovitis, and effusion on fluid, are consistent with active inflammatory changes located in hips with AS [26].

Our study showed excellent reliability among four raters in AS patients. The intrarater ICC was 0.993 and interrater ICC was between 0.955 and 0.977. The results were in accordance with previous studies applied on hip OA [24, 25] and SpA [26]. Zheng et al.[26] reported that the reliability of HIMRISS improved from 0.67 to 0.90 after two training sessions in cases with SpA. The reliability of detecting femoral BML, acetabular BML and synovitis effusion was very good after the two exercises (the overall ICC was 0.73, 0.84 and 0.88, respectively).

The study conducted by Zheng et al. [26] showed that it was more notable to see the correlations between disease activity and HIMRISS from bilateral rather than unilateral hip joint scores. The sum HIMRISS correlated moderately with ASDAS‐CRP (*r* = 0.67, *p* < 0.001) and CRP (*r* = 0.55, *p* < 0.01) and weakly with ASDAS‐ESR (*r* = 0.48, *p* < 0.001). Unfortunately, the study did not discriminate the degree of radiographic hip involvement and as a result failed to confirm the clinical advantage of HIMRISS in detecting early hip inflammation lesions in SpA patients. In our series, there was a significant correlation of HIMRISS with BASDAI (*r* = 0.540, *p* < 0.001), BASFI (*r* = 0.581, *p* < 0.001), ASDAS-ESR (*r* = 0.604, *p* < 0.001), ASDAS-CRP (*r* = 0.575, *p* < 0.001), ASQOL (*r* = 0.478, *p* < 0.001), and HHS (*r* = -0.468, *p* < 0.001). These results were also consistent with the research done by Zheng et al. [26]. Noteworthy in terms of this correlation is the relatively weak correlation between HIMRISS and HHS. As a hip joint-specific parameter, HHS demonstrated a relatively weak correlation with HIMRISS(*r* = -0.468), compared with BASDAI (*r* = 0.540), BASFI (*r* = 0.581), ASDAS-ESR (*r* = 0.604), and ASDAS-CRP (*r* = 0.575). Our explanations are as follows: The HHS is an observational assessment that consists of eight questions and a physical examination. The questions were divided into three categories: pain (0–44 points), function (0–47 points), and level of activity. Although HHS is less sensitive to a patient’s subjective bias, it does not account for individual differences, such as age, comorbidities, or problems from the spine or other joints that may impact the score. For example, the scores from function status and level of activity may suffer from syndesmophytes and ankylosis of the spine.

Moreover, we classified hips into no, mild and moderate to advanced hip involvement subgroups according to HHS of involved hip and BASRI-hip score. Traditionally, the severity and progression of hip involvement in patients with AS are judged only by radiographic presentations, such as the BASRI system. However, clinical hip involvement, including symptoms and functional status, is not taken into consideration. Hence, in our study, we introduced a grouping strategy in combination with clinical and radiographic evaluations, which was valuable in testing the validity of HIMRISS. We classified these hips into no hip involvement group (HHS ≥ 80 and BASRI ≤ 1), mild hip involvement subgroup (BASRI = 2 or BASRI ≤ 1 and HHS ≤ 79), and moderate to advanced hip involvement subgroup (BASRI ≥ 3). To further detect early structural damage of the hip, we added HHS as grouping criteria. Once radiographic hip involvement progresses to the stage of BASRI ≥ 3, it should be regarded as moderate to advanced, regardless of the HHS value. The mild hip involvement subgroup had a significantly higher HIMRISS than the no hip involvement subgroup (29.38 vs. 14.50%, *p* = 0.009). This result can be considered further proof that HIMRISS is a reliable imaging tool to detect early structural damage in patients with AS.

The main limitation to our study came from the nature of its single‐center cross-sectional research. Relatively few participants took part in the study, which may have negatively impacted the evaluation of the rate and degree of hip involvement in patients with AS.

In conclusion, HIMRISS applied in patients with AS demonstrated a satisfying reliability, and it was in significant clinical association with a series of AS specified clinical and laboratory parameters. And there was a particular concern that the hips with mild hip involvement had a significantly higher HIMRISS than the ones without hip involvement. HIMRISS is a reliable quantitive assessment tool for evaluating early hip involvement in patients with AS.

## Conclusions

The application of HIMRISS in patients with AS demonstrated a satisfactory reliability, which means it can be a reliable quantitive assessment tool for evaluating early hip involvement in patients with AS.

## Data Availability

The datasets used and/or analyzed during the current study are available from the corresponding author on reasonable request.

## Abbreviations

AS: Ankylosing spondylitis
ASDAS: Ankylosing Spondylitis Disease Activity Score
ASQoL: Ankylosing Spondylitis Quality of Life
AP: Anteroposterior
BASDAI: Bath Ankylosing Spondylitis Disease Activity Index
BASFI: Bath ankylosing spondylitis functional index
BASRI-hip: Bath Ankylosing Spondylitis Radilogy Index
BMI: Body Mass Index
BMLs: Bone Marrow Lesions
CRP: C reactive protein
DMARDs: Disease-modifying Anti-rheumatic Drugs
EAMs: Extra-articular Manifestations
ESR: Erythrocyte sedimentation rate
HHS: Harris Hip Scoring
HIMRISS: Hip Inflammation MRI: Scoring System
HLA: Human leukocyte antigen
ICCs: Intraclass Correlation Coefficients
MRI: Magnetic resonance imaging
NSAIDs: Non-steroidal anti-inflammatory drugs
OA: Osteoarthritis
PROs: Patient-reported Outcomes
SF-12: Short Form-12
SpA: Spondyloarthritis
THA: Total Hip Arthroplasty
T1 TSE: T1-Weighted Spin Echo
